# Potential benefits of a blend of essential oils on metabolism, digestibility, organ development and gene expression of dairy calves

**DOI:** 10.1038/s41598-023-30088-y

**Published:** 2023-02-28

**Authors:** Joana P. Campolina, Sandra Gesteira Coelho, Anna Luiza Belli, Luiz F. Martins Neves, Fernanda S. Machado, Luiz G. R. Pereira, Thierry R. Tomich, Wanessa A. Carvalho, Raquel M. P. Daibert, Daniele R. L. Reis, Suely F. Costa, Alessandra L. Voorsluys, David V. Jacob, Mariana M. Campos

**Affiliations:** 1 Departamento de Zootecnia, Escola de Veterinária, Universidade Federal de Minas Gerais, Belo Horizonte, Minas Gerais 30161-970 Brazil; 2 Embrapa Gado de Leite, Empresa Brasileira de Pesquisa Agropecuária (EMBRAPA), Juiz de Fora, MG 36038-330 Brazil; 3 Departmento de Medicina Veterinária, Universidade Federal de Lavras, Lavras, Minas Gerais Brazil; 4 Adisseo, Campinas, São Paulo Brazil

**Keywords:** Nutrition, Animal physiology, Immunology, Gastroenterology, Health care

## Abstract

The objective of this study was to evaluate blood cells and metabolites, insulin-like growth factor-1 (IGF-1), digestibility, internal organs weight and histology, gene expression, and spleen cell proliferation of pre-weaned bull calves supplemented with a blend of essential oils in milk replacer (MR). Sixteen newborn Holstein × Gyr crossbred dairy bull calves, with body weight at birth of 33.3 ± 3.7 kg, were housed in individual sand bedded pens, blocked by genetic composition, and randomly assigned to 1 of 2 treatments in a randomized complete block design: Control (CON, n = 8) and blend of essential oils supplementation (BEO, n = 8, 1 g/day/calf, Apex Calf, Adisseo, China). The commercial blend was composed by plant extracts derived from anise, cinnamon, garlic, rosemary, and thyme. Animals were fed 5 L of MR/day reconstituted at 15% (dry matter basis), divided into two equal meals. Water and starter were provided ad libitum. ß-hydroxybutyrate, urea, and glucose were evaluated weekly, IGF-1 was evaluated biweekly, and total blood cell count was performed every four weeks until the end of the trial at eight weeks of age. Feed samples were collected three times a week and polled for weekly analysis. Apparent total nutrient digestibility was determined from d 56 to 60 of age. On d 60 ± 1, animals were euthanized for organ weight, histology, spleen cell proliferation, and intestinal gene expression analysis. Data were analyzed independently using linear mixed models using the REML method in the nlme package in R for continuous outcomes. A non-parametric test was used for ordered categorical outcomes using the Artools package in R. There were no differences between groups for blood evaluations, digestibility, gene expression, and a spleen cell proliferation assay. However, BEO calves presented a heavier pancreas, heavier intestines, bigger ileum villi, and higher cecum butyrate levels (*P* < 0.05), demonstrating that the EO supplementation helped intestinal development and symbiotic bacteria. It was also observed in CON animals’ heavier respiratory tract and a higher eosinophil count (*P* < 0.05). Therefore, the organs where eosinophils are more active had a better response for BEO animals. No differences were found in the intestinal gene expression in the immune context. These results demonstrate that supplementing essential oils in MR could contribute to gut development and immune function. However, more research is needed to understand its impact on body development and define the best dosage and route of administration.

## Introduction

The use of antimicrobials as growth promoters in livestock has been questioned lately, particularly because of the possibility of creating bacterial resistance and one health concept^1–3^. Antimicrobials used to treat farm animals, especially neonatal diseases, have been a concern since they are used the same drugs as those used in human medicine^2,4^. Moreover, incorrect use of antimicrobials to prevent or treat diseases could increase the pathogens' resilience and weaken the host immune system through gut dysbiosis ^6,7^. It must also be pointed out that animal welfare correlates with animal health and antimicrobial use in dairy farms, an item measured to assess animal condition and wellbeing^5^. Therefore, the politics of antimicrobial use to treat diseases in dairy farms and the rationalization of its use are in constant update by several national veterinary organizations^1^.

The pre-weaning period is the phase in a dairy farm with the highest mortality rates^4,5^. The calves still have an immature immune system and are susceptible to enteric and respiratory diseases^6^. The gastrointestinal tract (GIT) is the largest organ of the immune system^7^. Therefore, since intestinal microbiota has an important role in regulating immune responses outside of the gut, it is important to assure and improve good microbes colonization on this site^8^. The gut microbiome will be crucial to optimize calf performance and health^9^. However, once the ruminal and gut microbiome is settled and complete in an older animal, it is difficult to manipulate this ecosystem permanently^13^. That is why manipulating and developing the calf’s gut microbiota at a young age is important since it is a window of opportunity to mediate metabolism, growth, and immune response^9,10^.

Therefore, some dietary practices and additives could influence nutrient use and commensal microbiota homeostasis, and animals’ immune response, especially during early life^11–13^. Colostrum and transitional milk supplementation during the first days of age, ruminal transfaunation inoculation, supplementation of pre and probiotics are potential strategies used to manipulate and improve microbial colonization and gut development of the young calf, and consequently, improve its immune system^9,14^. Thus, feed additives have been in search as an alternative not only to enhance livestock performance but also for its anti-inflammatory, antimicrobial, ruminal modulation, antioxidant and immunological improvement^15^. Within the supplementation of feed additives that could be an option for growth promoters use, the lasted hot topic is essential oils (EO).

The EO are plant metabolites natural extracts with antibacterial, antiviral, antifungal, antioxidant, and anti-inflammatory activities^16,17^, beneficial for gut microbiota^18^ and calf performance^19^. Different plants are used to obtain EO, as well as different molecules, with different actions, in each of these oils^17,20^. Therefore, additives using a combination of these EO, or blends, have been tested lately to modify the ruminal ecosystem and microbiota, improve nutrient utilization, performance, and health during the early stages of life^9^. Previous results using different plants' EO through a liquid diet have shown potential benefits for calves' growth and health improvement^21^, still, there is limited information on its use in young ruminants.

This study aimed to evaluate the effect of a commercial blend of EO supplementation in milk replacer (MR) on immunity, nutrient digestibility, organ development, and gene expression in dairy bull calves during the pre-weaning phase. Performance and carry-over effects were evaluated in our previous work^22^ and were demonstrated in the present work with a descriptive purpose. We hypothesized that EO supplementation through liquid diet could enhance immune response, help gut development, and consequently, increase nutrient digestibility.

## Material and methods

Animal care and use protocol guidelines were strictly followed for this experiment, under protocol number 9078250118 approved by Embrapa (The Brazilian Agricultural Research Corporation) Dairy Cattle Ethics Committee. The Embrapa was established by the Brazilian Ministry of Agriculture, Livestock and Food Supply.

### Animals, management, and treatments

This study was conducted in Embrapa Dairy Cattle facilities (Coronel Pacheco, Brazil) from March to July 2018. Sixteen newborn Holstein and crossbred (Holstein × Gyr) bull calves with an average initial body weight of 33.3 ± 3.7 kg were separated from their mothers immediately after birth and used for this trial. They received 10% of their body weight of good quality colostrum (Brix > 23%) during the first six hours of life and had their umbilical cord immersed in a 10% iodine solution for the first three days of age. The bull calves were allocated in a barn with open sides, in individual sand-bedded pens (1.25 × 1.75 m) and tethered with 1.2 m long chains. Ad libitum water and commercial calf starter (Soymax Rumen pre-initial Flocculated, Total Alimentos, Três Corações, Brazil, Table 1) were provided during all experimental period since the first day of life.

**Table 1:** Nutrient composition (% DM basis ± SD) of milk replacer (MR) and starter.

Item	MR^a^	Starter^b^
DM (%)	96.0 ± 0.4	86.7 ± 0.7
CP (% of DM)	19.4 ± 0.5	17.1 ± 0.5
Ether extract (% of DM)	14.1 ± 0.6	3.9 ± 1.2
Organic matter (% of DM)	9.7 ± 0.2	7.2 ± 1.5
NDF (% of DM)	–	22.1 ± 2.9
ADF (% of DM)	–	10.6 ± 0.9
Gross energy (Mcal/kg of DM)	4.5 ± 0.1	4.3 ± 0.1

^a^Powder integral milk, wheat isolated protein, acidifying additive, whey, coconut oil, palm oil, vitamin A, Vitamin D3, Vitamin E, Vitamin C (Kalvolak, Nutrifeed, Netherlands).

^b^Basic composition: oats (rolled grains), calcitic limestone, sodium chloride, corn gluten meal, defatted corn germ, wheat bran, soybean meal, rice hulls, kaolin, molasses, flocculated corn, ground corn, corn grain, alfalfa hay, monensin, citrus pulp, dried sugarcane yeast, whole toasted soybean, sodium selenite, copper sulfate, manganese sulfate, cobalt sulfate, iron sulfate, zinc sulfate, calcium iodate, vitamin A, vitamin B1, vitamin B12, vitamin B2, vitamin B6, vitamin C, vitamin D3, vitamin E, vitamin K, niacin, pantothenic acid, folic acid, biotin, propionic acid, caramel aroma, milk aroma, and probiotic additive.

A liquid diet was provided twice a day (0800 and 1600 h) in buckets provided with rubber teats (Milkbar, New Zeland). At d 2 and 3 of life, calves received 5 L/day of transition milk divided equally into two meals, and from the 4 to 60 days, they were fed with 5 L/day milk replacer divided equally in two meals (MR, Kalvolak, Nutrifeed, Netherlands; Table 1), reconstituted to provide 15% of total solids, 194 g of crude protein and 60 g of fat. The passive immune transfer was checked on d 3, where a serum sample was collected via jugular venipuncture. Tubes were left at room temperature for 30 min and then centrifuged at 1800×*g* for 10 min (22–25 °C). After centrifugation, the serum was evaluated in a Brix refractometer (Aichose refractometer, Xindacheng, Shandong, China). Calves were enrolled only if the Brix was higher than 8.4%.

On day 4, bull calves were randomly assigned to one of two treatments, following: (i) control (CON, no additive; n = 8) and (ii) blend of essential oils supplementation (BEO, 1 g/day/calf, Apex Calf, Adisseo, China; n = 8). The month of birth, weight, and Brix were checked during the assignment to ensure that both treatments were balanced. Apex calf (Apex Calf, Adisseo, China) is a commercial additive that contains a blend of plant extracts derived from anise, cinnamon, garlic, rosemary, and thyme. This additive was incorporated in the MR during the experiment following manufacturer recommendations. The amount of the additive for each meal was weighed previously and kept in 15 mL tubes in a dark box. This amount was mixed with 10 mL of MR, homogenized, and incorporated in 0.49 L of MR (0.5 g/calf at morning meal and 0.5 g/calf at afternoon meal) to ensure total ingestion of the product. As soon as the animal finished ingesting 0.5 L MR with 0.5 g of the additive, the bucket was refilled with the rest of the MR.

### Intake, performance, and growth

Feed intake (MR, starter, and water), performance, and body frame development were measured between 4 and 60 d of age with a descriptive purpose. The feed intake was calculated daily by subtracting the refusals from the provided amount. Samples of MR and starter were collected three times a week to obtain a weekly pool for nutrient analysis.

The body weight (BW) and body frame development (wither height (WH), rump height (RH), and heart girth (HG)) were measured weekly before the morning meal, using a weighing machine (ICS 300, Coimma, Dracena, Brazil), a portable hypometer and a measuring tape.

### Nutrient apparent digestibility and nutrition composition analysis

Feed digestibility was conducted during the last five days of the trial, between d 56 and 60 of age. A rubber mat (WingFlex, Kraiburg TPE GmbH & Co., Waldkraiburg, Germany) was placed on each individual stall to allow daily fecal collection. Feces were collected and weighted daily from d 56 to 60 and frozen at − 20 °C for further analysis. On d 59 animals were transferred to metabolic cages (1.5 × 0.8 m, Intergado Ltda., Contagem, Brazil) for 24 h urine collection and the last day of fecal sampling. The flask that stored the urine during the trial was placed in a cooler covered with ice to avoid bacteria growth and nitrogen loss. After the collection period, the urine’s total volume, weight, and density were recorded, and a sample was frozen at − 20 °C for further analysis. During the digestibility trial, MR, starter, and refusals samples were collected and pooled for the five days and stored and frozen at − 20 °C for further analysis.

Starter and MR samples were collected weekly and during the digestibility trial. The feces collected during digestibility were oven-dried at 55 °C for 72 h and ground in Wiley mill (model 3, Arthur H. Thomas Co., Philadelphia, PA) through a 1-mm screen for analysis. They were analyzed to determine dry matter (DM, Method 934.01), crude protein (CP, Method 988.05), ether extract (EE, Method 920.39), ash (Method 942.05), according to AOAC^23^. The concentrations of neutral detergent fiber (NDF) and acid detergent fiber (ADF) were determined in sequence using the method described by Van Soest et al.^24^. Gross energy was determined using an adiabatic bomb calorimeter (Parr Instrument Company, Moline, IL).

Apparent digestibility of each nutrient (%) was determined considering nutrient intake (NI) and nutrient feces recovery (NFR) using the formula:

$$\frac{NI-NFR}{NI}\times 100$$

Nitrogen balance was determined by the difference between nitrogen intake (NI) and nitrogen fecal (NF) and urinary nitrogen (NU) using the formula:

$$NI-(NFR+NU)$$

Gross energy intake (GEI) was determined by the difference between gross energy of the diet provided (starter gross energy (GES) and MR gross energy (GEMR)) and refusals gross energy (GER) using the formula:

$$\left(GES+GEMR\right)-GER$$

Digestible energy intake (DEI) was determined by the difference between GEI and energy fecal excretion (GEF). To determine metabolizable energy intake (MEI), the energic losses from the urine (GEU) were subtracted from DEI.

### Blood sampling

To obtain a baseline, jugular blood samples were collected at birth before colostrum ingestion. After that, there was a weekly collection 3 h after morning feeding to obtain the serum concentrations of beta-hydroxybutyric acid (BHB), serum urea with tubes without anticoagulant, plasmatic glucose with sodium fluoride tubes and, biweekly, for plasmatic IGF-1 with heparin tubes (Labor Import, Osasco, Brazil). Tubes were centrifuged at 3000 × *g* for 10 min at room temperature (22–25 °C), and duplicates of each sample were individually allocated into microtubes and frozen at − 20 °C for further analysis. The serum concentration of BHB and urea were determined by an auto-analyzer (Cobas Mira Plus, Roche Diagnostic Systems, Risch-Rotkreuz, Switzerland) using commercial kits (Ranbut-D-3-Hidroxibutyrate, Randox Laboratories Ltd., Antrim, UK; Urea UV, Kovalent do Brasil Ltda., Bom Retiro São Gonçalo, Brazil). Plasma glucose was measured in a microplate Spectrophotometer EON (Biotek Instruments Inc., Winooski, VT) using the enzymatic colorimetric method (Kovalent do Brasil Ltda., Rio de Janeiro, Brazil). Plasma IGF-1 concentrations were analyzed using a chemiluminescence assay (Immulite2000 Systems 1038144, IGF-1 200, Siemens Healthcare Diagnostics Products Ltd., Llanberis, Gwynedd, UK).

On days 0, 30, and 60, blood samples were collected for complete blood count by jugular vein puncture into EDTA tubes (Labor Import, Osasco, Brazil) and immediately transported on ice to the laboratory. An automatic hematology cell counter (SDH – 3 vet, Labtest Diagnóstica S.A., Brazil) was used to evaluate: red blood cell count (RBC), packed cell volume (PCV), hemoglobin (Hb), mean corpuscular volume (MCV), mean corpuscular hemoglobin concentration (MCHC), platelet and total white blood cell count. Manual white cell blood differential counting was also performed by microscopic examination evaluating 100 leukocytes in a 1000 × microscopic magnification for total leukocyte count, basophils, eosinophils, neutrophils, band neutrophils, segmented neutrophils, lymphocytes, and monocytes. Morphological changes, such as toxic neutrophils, reactive lymphocytes, and activated monocytes, were calculated. With the previous results calculated platelet to lymphocytes ratio (PLR) and neutrophils to lymphocytes ratio (NLR). The PRL and NRL are novel inflammatory markers and were chosen to verify if they could be applied as biomarkers to predict inflammation and mortality^25^ and balance between inflammation and adaptive immunity to predict disease course as already done in human medicine^26^.

### Comparative slaughter and histology

All bull calves were euthanized on day 60 ± 1 to compare internal organs development using the procedures recommended by the Brazilian Federal Veterinary Medicine Council^27^. Immediately after stunning and slaughtering, the jugular was cut to drain the body's circulating blood. The abdominal cavity was then opened, and each region of the gastrointestinal tract was isolated and tied off. Internal organs and body parts were removed and weighted following the order: spleen, bladder, all intestinal tract, liver, pancreas, omentum, perirenal fat, kidney, pre stomachs (rumen-reticulum, omasum), abomasum, small and large intestine, tongue, heart, lungs + trachea. After this, the organs with biological content were emptied and weighted again (bladder, rumen-reticulum, omasum, abomasum, small and large intestine). The weight of the organs was evaluated in proportion to the weight of the empty animal; thus, the animal's fluids' weight was subtracted from the animal’s live weight. The length of small and large intestines was measured using a metric tape. Ruminal and cecal fluid samples were immediately collected to measure pH, VFA, and NH_3_-N. After these procedures, some parts were then emptied and then weighted again.

Approximately 9 cm^2^ area samples were collected for comparative histology: rumen ventral sac, rumen dorsal sac, omasum laminae, abomasum, duodenum (ten centimeters under the abomasum), ileum (40 cm before the ileum-cecum junction), and colon (40 cm after the ileum-cecum junction). Tissue samples were immediately placed in flasks with formalin for fixation. Forty-eight hours after fixation, formalin was replaced by 70% alcohol and protected from the light. The samples were processed to include paraffin and then sectioned in 5 µm thickness using a manual microtome (Olympus CUT 4055, Tokyo, Japan). For morphometric analysis, sheets were colored using hematoxylin–eosin. Images were captured using a light microscope (Olympus CX31, Tokyo, Japan), connected to a camera (Olympus OSIS SC30, Tokyo, Japan), using Cell-B software (Olympus, Tokyo, Japan). AxioVision 4.8.2-06/2010 (Carl Zeiss Images Systmes®237, Jena, Germany) was used for morphometric interpretations. For rumen and omasum samples, papilla`s area, height, and mitotic index (MI) of the epithelium basal layer were analyzed. For MI determination, 2000 basal layer cells were counted using a light microscope. Estimation considered the ratio between the number of cells in the mitotic division and the total counted cell number^28^. The height (μm) and area (μm^2^) of villi in the duodenum and ileum regions; the depth (μm) of gastric fossets and crypts in the duodenum, ileum, and colon regions were measured. Cell proliferation was determined by the count of mitotic figures in the epithelium of the gastric and intestinal glands in 10 fields, with an increase of 400x.

### Ruminal and cecum pH and ammonia nitrogen

Ruminal fluid samples were obtained on days 14, 28, 42, and 60, using an esophageal tube four hours after morning feeding. On the day of the euthanasia, samples were collected directly from the rumen and cecum of the animals. Rumen pH was immediately measured using a pH meter (Phmetro T-1000, Tekna, Araucária, Brazil). Ten milliliters of the filtrated ruminal fluid were then acidified with 2 mL of 20% metaphosphoric acid for VFA analyses and ten milliliters with 0.2 N 50% sulfuric acid for NH_3_-N analyses. These samples were stored at − 20 °C for further analysis. For NH_3_-N concentration it was used a colorimetric distillation method proposed by Chaney and Marbach^29^, where its absorbance was measured at 630 nm (Termo Fisher Scientific, Madison, USA) after Kjedahl destilation with magnesium oxide and calcium chloride. The VFA ruminal concentrations were measured by gas chromatography. They were thawed and centrifuged at 13,000 rpm, for 15 min, at 13 °C. The supernatant was collected, filtered, and analyzed as previously described^22^.

### Splenocyte proliferation assay

The spleen function combines the innate and adaptive immune response, and removes older erythrocytes, microorganisms, and cellular debris from the circulation, being the most important organ for antibacterial and antifungal immune reactivity^30^. To evaluate cell proliferation to bacterial antigens, immediately after the animal slaughter and isolation of the organs, the spleen had its measurements taken and placed into ice to be processed shortly.

Five grams of the tissue were ground at the lab, followed by density gradient centrifugation on a Ficoll-Hypaque solution at 400 g for 30 min (Sigma, USA) for mononuclear cell isolation. The splenocyte cells (5 × 10^6^ cells/well) were seeded in flat-bottomed micro-culture plates and stimulated with lipopolysaccharide from *E. coli* (10 ng/mL; Sigma, USA), or Phorbol 12-myristate 13-acetate (PMA; 25 ng/mL), or *E. coli* B41 lineage extract (20 ng/mL) from streptomycin-resistant derivate of bovine ETEC strains isolated according to Smith and Halls^31^. The isolated colony of *E. coli* B41 lineage were lysate and diluted in PBS buffer added with a cocktail of protease inhibitors (Protease Inhibitors Set, Sigma, USA). *E. coli* and LPS were chosen since *E. coli* is one of the most important mediators of calf diarrhea in the first weeks of life^32^. Therefore, it could be a good choice to visualize the indirect effects of the EO on cell proliferation.

The mononuclear cells from the spleen were then cultured in RPMI 1640 medium (Sigma, USA) with 10% heat-inactivated fetal calf serum (Gibco, USA), 2 mM L-glutamine (Sigma, USA), 100 µg/mL of streptomycin, and 100 U/mL of penicillin (Sigma, USA) at 37 °C in a 5% humidified CO2. Cell proliferation was analyzed by MTT assay (3-(4,5-dimethylthiazoyl)-2,5diphenyltetrazolium Bromide; Sigma, USA) according to fabricant instructions (Sigma) using non-stimulated cells as a negative control. Briefly, the stimulated splenocytes were incubated at 37 °C in a 5% humidified CO_2_ incubator for 48 h. Ten µL of MTT (5 mg/mL) were added to each well afterward, and incubation was carried out for 4 h at 37 °C. The supernatants were aspirated carefully, and 150 µL of DMSO was added to each well. The plates were shaken for an extra 10 min, and the absorbance values were read at 570 nm with an ELISA reader. The absorbance values were compared among stimulated and non-stimulated groups.

### Gene expression and RT-qPCR

Gene expression analyses from buffy coat cells, ilium, and colon biopsies were performed by RT-qPCR. Briefly, peripheral whole blood from CON (n = 8) and BEO (n = 8) groups was collected on days 30 and 60 and centrifuged at 800 g for 10 min at room temperature for buffy coat isolation. The white blood cells and platelets (buffy coat) formed a layer on red blood cells that were carefully removed with a micropipette. According to the fabricant instructions, red blood cells were then lysate by Ammonium-Chloride-Potassium Lysing Buffer (ACK; Thermoscientific, Waltham, USA), and only the white layer of cells was frozen at RNA protect reagent (Qiagen, Hilden, Germany) until RNA extraction. The ileum and colon biopsies were obtained from the animal’s necropsy and kept on RNAprotect reagent (Qigen, Hilden, Germany) until analysis. The RNA extraction from buffy coat and organ samples was performed with RNeasy Mini kit (Qiagen, Hilden, Germany). The obtained total RNA was quantified by the Nanodrop 1000 Spectrophotometer (Thermo Scientific, Waltham, USA), and the cDNA synthesis was performed by SuperScript III First-Strand kit (Thermo Scientific, Waltham, USA), all according to the manufacturer's instructions^33^.

The RT-qPCR assays occurred in 7500 Fast Real-Time PCR System (Thermo Scientific, Waltham, USA), using the PowerUp SYBR Green Master Mix (Thermo Scientific, Waltham, USA) to verify the expression of interleukin 6 (IL-6) and interleukin 10 (IL-10) genes. It was used β-actin, GAPDH, and Ubiquitin as reference genes based on expression stability calculated with RefFinder online software. Each sample calculated the average of Ct values from targets and reference genes using ABI Real-Time PCR 7500 software v.2.3 (Thermo Scientific, Waltham, USA).

### Statistical analysis

Statistical analysis was conducted utilizing R® (R Core Team, 2019). A randomized complete block experimental design with repeated measures was implemented to test the hypothesis of the effect of the BEO on each performance outcome. Animals were blocked by genetic composition as ¾ or 5/8 Holstein × Gyr crossbreds. The outcomes analyzed were nutrient digestibility, organ weight, histology, ruminal, cecum, and blood parameters, and gene expression. For each treatment was assigned eight experimental units were assigned.

Each outcome was analyzed independently using linear mixed models (package: nlme). Each independent outcome was modeled as a function of the following fixed effects: treatment, experimental week, and the interaction between treatment and week. Birth weight and serum Brix value were tested as a covariate but did not improve statistical significance. Therefore, they were eliminated from the model. The genetic composition of the animal was included as a blocking effect. The effect of bull calf within treatment was included in the models to account for individual variability. All outcomes were tested for homogeneity of variance and normality to meet the required assumptions of this model using residuals versus fits and Q-Q plots, respectively. A variable transformation using Box-Cox was applied to milk replacer intakes to meet the assumption. A 95% Confidence Interval was adopted for all the tests.

The continuous outcomes such as intakes, structural growth, performance, ruminal, and blood parameters were analyzed with ANOVA. *P*-values were produced with a Fisher test and estimated marginal means and SEM were calculated with the emmeans package. The categorical outcomes fecal and respiratory scores were analyzed using a non-parametric aligned rank transformation test implemented in the R package ARTool.

The outcomes that had a single measure during the study, such as nutrient digestibility, nitrogen balance, energy partitioning organ/viscera weight and size, organ histology, splenocyte proliferation, and gene expression, were analyzed using the linear mixed model (package nlme) were calf was the random effect and treatment was the fixed effect.

## Results

### Intake, performance, and body measurements

The feed intake, performance, and body measurements were evaluated for descriptive purposes and revealed no differences within treatments. The average passive immune transfer Brix value was 10.4 ± 1.0. The initial and final weights were 33.3 ± 3.7 kg and 66.1 ± 4.5 kg respectively, with an average growth of 11.3 ± 3.0, 12.1 ± 3.2, and 17.8 ± 3.7 cm for WH, RH, and HG respectively. Animals had an average starter and water intake of 0.27 ± 318 kg/day and 1.29 ± 980.5 kg/day respectively. The MR intake was 0.75 g/day with a decrease on weeks 2 and 3 to 0.71 ± 0.022 g/day due to diarrhea occurrence.

### Ruminal and cecum pH, VFA, and ammonia nitrogen

Ruminal pH presented lower values for the BEO treatment when compared to CON (*P* = 0.02, Table 2). A week effect was also observed, with a decrease of 14% in pH values from week 3 to week 9 for both groups. There were no treatment differences for the ruminal ammonia nitrogen and all VFA measured. However, as observed on pH, there was also a week effect for the VFA and C2:C3 proportions (*P* < 0.01, Table 2), with increasing values of all VFA and decreasing values of C2:C3 as the animals were growing older. The C2:C3 proportion also presented a treatment × week interaction, where it was observed 28% and 16% higher values for BEO animals on weeks 3 and 5, respectively. For weeks 7 and 9, those values did to presented differences.

**Table 2:** Rumen and cecum mean values of pH, ammonia nitrogen (Ammonia-N), and volatile fatty acids (VFA) of control bull calves (CON) and bull calves supplemented with essential oils blend (BEO) in milk replacer from 4 to 60 days of age.

Item	Treatment^a^		SEM	*P* value^b^		
	CON (n = 8)	BEO (n = 8)		T	W	T × W
Rumen pH	6.35	5.91	0.45	0.02	0.05	0.95
Rumen ammonia-N (mg/dL)	13.82	16.61	6.85	0.22	0.47	0.06
Rumen VFA (µmol/mL)						
Acetic (C2)	27.90	23.28	7.94	0.36	0.01	0.59
Propionic (C3)	23.07	18.21	8.12	0.37	< 0.001	0.68
Butyric (C4)	3.42	4.18	1.96	0.28	0.006	0.15
C2:C3	1.40	1.55	0.27	0.34	< 0.001	0.02
Cecum pH	7.25	7.24	0.27	0.93	–	–
Cecum ammonia-N (mg/dL)	9.7	9.5	1.2	0.89	–	–
Cecum VFA (mol/100 mol)						
Acetic (C2)	19.79	24.30	3.74	0.17	–	–
Propionic (C3)	12.26	12.77	2.57	0.81	–	–
Butyric (C4)	1.89	3.33	0.67	0.05	–	–
C2:C3	1.77	1.98	0.35	0.43	–	–

^a^CON = control; BEO = 1 g/calf/day blend of essential oil.

^b^T = treatment effect; W = week effect; T × W = treatment by week interactions.

Cecum parameters were evaluated only on the last day of the trial, after euthanasia. There were no differences within treatment groups for all evaluated parameters (*P* > 0.05, Table 2), with an exception for butyric acid values, which presented values 76% higher for the BEO group (*P* = 0.05, Table 2).

### Blood sampling

There were no differences within treatments for all metabolic—BHB, urea, and glucose—and hormonal—IGF-1—parameters (*P* > 0.05, Table 3). However, all these parameters presented a week effect (*P* < 0.01, Table 3), increasing concentration values as the animals grew older. As for the hemogram, there was only a difference in red blood cell size through the weeks, with a decrease in MCV from week 1 to 9 (*P* = 0.04, Table 3). A treatment effect was observed for the eosinophils count for the white blood cell count, with 2.4 times lower values for the BEO group (*P* = 0.04, Table 3). As for the week effect on the white cell count, age impacted eosinophil count, segmented neutrophils count, lymphocytes count, PLR, and NLR, observing differences from week 1 to 9. There was a significant interaction of treatment × week for segmented neutrophils (*P* = 0.04), where BEO animals had 50% more cells on week 5 when compared to CON animals, but no differences on the other weeks.

**Table 3:** Blood concentrations of metabolites, insulin growth factor type 1 (IGF-1), and hematological parameters of control bull calves (CON) and bull calves supplemented with a blend of essential oils blend (BEO) in milk replacer from 4 to 60 days of age.

Item^a^	Treatment^b^		SEM	*P* value^c^		
	CON (n = 8)	BEO (n = 8)		T	W	T × W
BHB (mmol/L)	0.10	0.06	0.13	0.32	0.001	0.16
Urea (mg/dL)	12.14	12.59	0.10	0.87	0.01	0.48
Glucose (mg/dL)	96.57	96.52	18.40	0.99	< 0.001	0.93
IGF-1 (ng/mL)	96.47	92.70	35.8	0.79	< 0.001	0.11
RBC (× 10^6^/µL)	7.91	7.67	0.81	0.39	0.34	0.06
PCV (%)	34.70	35.29	4.37	0.69	0.79	0.07
Hb (q/dL)	10.73	10.98	1.35	0.57	0.87	0.07
MCV (fL)	44.25	44.28	3.70	0.11	0.04	0.61
MCHC (%)	31.31	31.09	1.45	0.63	0.25	0.51
Total leukocytes (/µL)	9999.03	11,288.81	3010.00	0.38	0.15	0.43
Basophils (/µL)	0.00	0.00	0.00	1.0	1.0	1.0
Eosinophils (/µL)	101.70	42.80	4.97	0.04	0.002	0.47
Band neutrophil (/µL)	21.91	27.08	0.05	0.16	0.71	0.47
Segmented neutrophils (/µL)	4840.68	5997.62	2380.0	0.17	< 0.001	0.04
Lymphocytes (/µL)	4790.32	5224.41	1160.0	0.75	< 0.001	0.61
Monocytes (/µL)	411.07	505.18	313.0	0.27	0.12	0.57
Platelet (× 10^3^/µL)	400.78	396.31	85.9	0.87	0.24	0.16
Plasmatic protein (g/dL)	5.80	6.12	0.66	0.26	0.17	0.42
PLR	0.12	0.09	0.04	0.16	< 0.001	0.22
NLR	1.88	1.87	1.65	0.99	< 0.001	0.78

^a^RBC, red blood cell; PCV, packed cell volume; Hb, hemoglobin; MCV, mean corpuscular volume; MCHC, mean corpuscular hemoglobin concentration; PLR, platelet lymphocyte ratio; NLR, neutrophils lymphocytes ratio.

^b^CON = control; BEO = 1 g/calf/day blend of essential oil.

^c^T = treatment effect; W = week effect; T × W = treatment by week interactions.

### Nutrient apparent digestibility and nitrogen balance

Total tract apparent digestibility and nitrogen balance were performed at the end of the trial from d 56 to 60. The digestibility of DM, OM, gross energy, CP, and EE did not differ among treatments (*P* > 0.05, Table 4). Outcomes related to nitrogen balance also presented similar values between treatments (*P* > 0.05, Table 4).

**Table 4:** Apparent nutrient digestibility % and nitrogen balance of control bull calves (CON) and bull calves supplemented with a blend of essential oils (BEO) in milk replacer from 4 to 60 days of age.

Item	Treatment^a^		SEM	*P* value^b^
	CON (n = 8)	BEO (n = 8)		T
Dry matter (g/day)	877	892	3.47	0.48
Organic matter (g/day)	914	926	2.59	0.64
Crude protein (mg/day)	908	922	1.98	0.64
Ether extract (mg/day)	957	956	1.43	0.96
Ingested nitrogen (g/kg of MW^c^/ day)	2.09	2.06	0.02	0.81
Fecal nitrogen (g/kg of MW^c^/ day)	0.17	0.15	0.006	0.65
Urine nitrogen (g/kg of MW^c^/ day)	0.36	0.37	0.01	0.98
Retained nitrogen (g/kg of MW^c^/ day)	1.56	1.55	0.02	0.91

^a^CON = control; BEO = 1 g/calf/day blend of essential oil.

^b^T = treatment effect.

^c^MW = metabolic weight.

### Comparative slaughter and histology

Euthanasia was performed in the morning before feeding not to impact the final weight. There were no differences between treatments for empty body weight (*P* = 0.12, Table 5). Most of the evaluated organs were statistically similar between treatments, except for the pancreas, respiratory tract, and small intestines. The BEO animal’s pancreas was 30% heavier when compared to CON (*P* = 0.05, Table 5). The lungs and trachea were 11% heavier on CON animals when compared to BEO (*P* = 0.03, Table 5). Moreover, the small intestines were 16% heavier in BEO animals (*P* = 0.03, Table 5), besides no difference in the intestinal length.

**Table 5:** Empty body, internal organs weight (% of empty body), and intestinal length of control bull calves (CON) and bull calves supplemented with a blend of essential oils (BEO) in milk replacer from 4 to 60 days of age.

Item	Treatment^a^		SEM	*P* value^b^
	CON (n = 8)	BEO (n = 8)		T
Empty BW (kg)	55.2	51.5	0.49	0.12
Organ weight (% of empty BW)				
Omental fat	0.29	0.31	0.04	0.40
Mesenteric fat	0.43	0.43	0.08	0.85
Perirenal fat	0.42	0.41	0.07	0.82
Pancreas	0.09	0.12	0.02	0.05
Liver	2.44	2.13	0.36	0.19
Lungs and trachea	2.14	1.91	0.13	0.03
Spleen	0.72	0.67	0.15	0.60
Heart	0.72	0.70	0.06	0.66
Kidneys	0.59	0.50	0.06	0.80
Togue	0.50	0.49	0.04	0.52
Bladder	0.07	0.08	0.02	0.33
Rumen-reticulum	1.67	1.65	0.05	0.84
Omasum	0.29	0.24	0.06	0.28
Small intestine	2.89	3.35	0.33	0.03
Large intestine	0.97	0.92	0.15	0.62
Small intestine length (m)	21.52	22.77	2.79	0.47
Large intestine length (m)	3.59	3.40	0.23	0.18

^a^CON = control; BEO = 1 g/calf/day blend of essential oil.

^b^T = treatment effect.

There were no differences in gastrointestinal tract development and histology (*P* > 0.05, Table 6), except for ileum villus height. Animals from BEO presented a 25% higher villus when compared to CON (*P* = 0.02, Table 6).

**Table 6:** Gastrointestinal tract development of control bull calves (CON) and bull calves supplemented with a blend of essential oils (BEO) in milk replacer from 4 to 60 days of age.

Item	Treatment^a^		SEM	*P* value^b^
	CON (n = 8)	BEO (n = 8)		T
Rumen ventral sac				
Cell proliferation	10.8	18.2	0.936	0.10
Total cells	2012	2017	0.810	0.22
Mitotic index	0.005	0.009	0.0004	0.12
Papillae height (mm)	2.07	2.04	0.041	0.31
Papillae area (mm)	6.37	5.80	0.232	0.68
Rumen dorsal sac				
Papillae height (mm)	2.68	2.04	0.048	0.07
Papillae area (mm)	4.51	3.77	0.139	0.28
Omasum				
Cell proliferation	20.1	17.0	1.11	0.61
Total cells	2020	2017	1.98	0.61
Mitotic index	0.01	0.01	0.0005	0.61
Papillae height (mm)	0.39	0.48	0.013	0.25
Papillae area (mm)	0.11	0.17	0.071	0.15
Abomasum				
Fossette depth (mm)	0.27	0.27	0.006	0.96
Glandular depth (mm)	0.15	0.13	0.003	0.24
Cell proliferation	9.11	8.36	0.330	0.70
Duodenum				
Villus height (mm)	0.39	0.38	0.071	0.79
Villus area (mm)	0.57	0.57	0.013	0.94
Crypt depth (mm)	0.31	0.29	0.004	0.19
Cell proliferation	24.40	25.87	1.280	0.83
Ileum				
Villus height (mm)	0.25	0.31	0.006	0.02
Villus area (mm)	0.33	0.40	0.008	0.12
Crypt depth (mm)	0.29	0.29	0.004	0.99
Cell proliferation	44.87	50.05	2.390	0.69
Colon				
Cell proliferation	12.40	18.12	0.650	0.15
Crypt depth (mm)	0.35	0.37	0.002	0.31

^a^CON = control; BEO = 1 g/calf/day blend of essential oil.

^b^T = treatment effect.

### Splenocytes proliferation and gene expression

Splenocyte proliferation assay was performed to evaluate supplementation effect over cellular response to bacterial antigens. The spleen cells were stimulated, in vitro, with *E. Coli* antigen extract and lipopolysaccharide. To verify potential growth inhibition of EO, PMA was used as a cell proliferation activator via Protein Kinase C (PKC). However, there were no differences between splenocytes proliferation under all tested treatments (Table 7).

**Table 7:** Splenocytes proliferation assay of control bull calves (CON) and bull calves supplemented with a blend of essential oils (BEO) in milk replacer from 4 to 60 days of age.

Item^a^	Treatment^b^		SEM	*P* value^c^
	CON (n = 8)	BEO (n = 8)		T
Negative control	0.17	0.22	0.01	0.35
PMA	0.27	0.28	0.02	0.91
ECE	0.15	0.18	0.005	0.56
LPS	0.202	0.205	0.009	0.96
BrEEC	0.21	0.23	0.09	0.76
MTT control	0.12	0.14	0.06	0.70

^a^PMA = Phorbol 12-myristate 13-acetate; positive control of the proliferation; ECE = *E. coli* extract; LPS = *E. coli* lipopolysaccharide extract from Sigma) ; BrEEC = ECE control.

^b^CON = control; BEO = 1 g/calf/day blend of essential oil.

^c^T = treatment effect.

Gene expression was evaluated on white blood cells (buffy coat) at 30 and 60 days of age and ileum and colon at 60 days of age. These samples were chosen based on the previous statistical results of this paper and our previous results^22^, and the evaluated genes were interleukin 6 (IL-6) and 10 (IL-10) since they are related to inflammatory responses and immunity regulation after treatment with BEO^34,35^. There were no differences within treatments for relative gene expression of IL-6 and IL-10 in the buffy coat, ileum, or colon (*P* > 0.05, Table 8). The relative gene expression of IL-6 and IL-10 increased over time in the buffy coat, but it was not significant (*P* > 0.05, Table 8).

**Table 8:** Relative gene expression of interleukin 6 (IL-6) and interleukin 10 (IL-10) in control bull calves (CON) and bull calves supplemented with a blend of essential oils (BEO) in milk replacer from 4 to 60 days of age.

Item	Treatment^a^		SEM	*P* value^b^		
	CON	BEO		T	W	T × W
	(n = 8)	(n = 8)				
Ileum						
IL-6	31.1	31.0	0.258	0.96	–	–
IL-10	31.4	31.0	0.214	0.69	–	–
Colon						
IL-6	26.8	27.2	0.196	0.68	–	–
IL-10	27.3	27.7	0.114	0.52	–	–
Buffy coat						
IL-6	24.1	24.7	0.69	0.27	0.69	0.81
IL-10	23.6	24.8	0.95	0.15	0.57	0.35

^a^CON = control; BEO = 1 g/calf/day blend of essential oil.

^b^T = treatment effect; W = week effect; T × W = treatment by week interactions.

## Discussion

The research and use of alternatives to replace artificial additives have increased widely, especially after the antimicrobials as growth promoters have been a concern for animal production and public health^2,36,37^. The EO exhibit broad-spectrum antimicrobial properties, improving growth and health status in various species^38–40^. It has been already shown that EO supplementation to other species had a positive impact on improving GIT enzyme activity and helping preserve the gut environment^41^. However, there is still a small amount of data evaluating EO for young dairy calves and its impact on gut development and immune function. Thus, the present study aimed to take the EO research to a further step and quantify the impact of EO supplementation on organ development and immune function. Our major findings were the positive impact on pancreas and small intestine weight, ileum histology, and cecum butyric acid increase.

The intake and performance showed the same pattern obtained with the females in our previous work^22^. The main goal of calves’ rearing is to double the weight at weaning, which was achieved in this trial. However, intake and performance results for EO supplemented animals are controversial, and there is a lack of works that supplements natural additives through a liquid diet. On our trial, we worked with a 1.0 g/day dosage divided into two meals, by manufacturer recommendation.

When evaluating the impact of EO supplementation on the GIT, differences were found for ruminal and cecum parameters, where BEO animals presented lower ruminal pH, higher cecum butyric acid, and higher C2:C3 ruminal proportions on weeks 3 and 5. It is known that the concentrations of VFA in different parts of the GIT are related to the direct function of the local microbiota. Thus, additives that provide changes in ruminal and intestinal microbiota can lead to changes in VFA and lactic acid profile and, consequently, ruminal pH^17,42^. Previous work showed that EO supplemented calves had more beneficial microorganisms in the intestinal microbiota^18^, and that some EO have a possible neuronal activity, altering animal behavior^43^. Pre-weaned Jersey calves treated with green tea extract or oregano extract anticipate the onset of rumination in one week compared to the control group^44^. Additionally, studies on other species have shown that EO supplementation increased gastrointestinal symbiotic bacteria, known as butyrate producers^45,46^. Therefore, higher content of this AGV could enhance, gut development, and modulation of the immune response^47^. In ruminants, the peak concentration of VFA happens in the rumen, and the second-highest concentration is found in the cecum, where further digestion of the fiber occurs^48^. The cecum butyrate produced by the local microbiota serves as the primary energetic nutrient to colonocytes and regulates multiple functions of gut cells, including its gene expression, cellular differentiation, tissue development, immune modulation, oxidative stress reduction, and diarrhea control, thus GIT function. It is possible that the lower gut communicates with the forestomaches, which means that nutrients in the lower gut can cause subsequent adaptations in the forestomaches^49^. This theory could explain why the EO has given in the MR in our trial impact over ruminal outcomes. Technically, since EO was given through liquid diet, its ingestion would pass the esophageal groove and go to the omasum and abomasum. Therefore, it was expected that the EO should impact only gut development.

Additionally, the GIT senses the nutrient supply during the first weeks of life and communicates with other organs that contribute to digestion, such as the liver and pancreas^49^. This manipulation will also be important for the animal’s future performance in the herd. The gut, especially the ileum, has lymphoid nodules, also called Peyer’s patches, that have an important role as an “immune sensor,” helping to promote epithelial repair and activating inflammatory sensors, regulating homeostasis, and the presence of innate immunity immune cells in the gut^50^. This connection also explains the integration of gut health and its immune cells and their migration to other body sites, as occurs to the mammary gland through an entero-mammary pathway^51^. Therefore, with theses scientific evidence, it is safe to say that the gut plays an important role in the immune system, microbiota, and disease behavior. It stimulates the immune function and the development of a mucosal layer, facilitating nutrient absorption and microbial activity cross-talk^52^. In our companion work^22^, the BEO animals present lower values for fecal score, corroborating with the positive findings for the GIT in this present work.

Alongside with the chemical differences in GIT, in our work, the BEO animals had heavier pancreas and intestines, and higher ileum villus height, indicating that supplemented animals could have a better GIT function and nutrient digestibility. A heavier pancreas indicates increased activity in this organ with a higher production of enzymes and a more active metabolism^53^. It is known that diet can cause an effect on pancreas development and function^54^, as well as an impact on the GIT microbiota and consequently this organ function^55^. Increased secretion of pancreatic enzymes implicates intestine adaptation to use the enzymes and corroborates with differences found in cecum butyrate. Additionally, knowing that a heavier intestine could be due to water content or cell proliferation, the histological differences found on the ileum indicate that the heavier intestines were due to higher cell content, influencing tissue absorption. Thus, BEO animals had a dietary effect of the EO, impacting positively on pancreas weight, intestinal development, and metabolism. Therefore, differences on nutrient digestibility were expected, but not found.

The digestibility results found in this trial were within the normal range previously reported^56–58^. Contrary to what we expected, both groups had similar digestibility. It was previously described that different inclusions rates of oregano EO tested on in vitro digestibility showed that high inclusions could be detrimental to digestibility and ruminal parameters. However, median inclusions could beneficially modify ruminal parameters, local microbiota and increase nutrient digestibility^59^. When tested in lactation cows, supplementation of oregano leaves did not change ruminal parameters nor apparent nutrient digestibility but decreased DMI and increased feed efficiency^60^. For young calves, including a combination of EO and prebiotics in a pelleted calf starter increased total tract digestibility for DM, CP, ADF, NDF, starch, and minerals^56^. Also, when EO was supplemented with monensin in the starter, EO demonstrated a greater impact on total nutrient digestibility, with a synergic effect when supplemented with monensin^57^. The lack of differences could be due to the supplementation route, the dosage, other additives, and interactions between them, or because animals were not nutritionally challenged. It is also important to remember that when digestibility was proceeded in this trial—at the 8th week, calves had a more developed rumen, and ate more starter. Thus, the nutrient intake proportion was higher from the starter. As shown before, there is an effect on starter form and carbohydrate source on the total digestibility tract^58^. The EO in this trial was supplemented via a liquid diet, thus a feed with higher digestibility and passage rate. Besides, no beneficial or detrimental effects over digestibility were found in our trial, to understand better the EO supplementation on GIT development and its impact on nutrient digestion and absorption, digestibility trials should be done around health events, as well animals should be nutritionally challenged.

However, besides no differences in nutrient usage, the EO supplementation could help control and maintain normal gut microbiota would consequently help the calf’s development. Previous studies in humans have shown that EO can regulate factors involved in the inflammation pathway, such as tumor necrosis factors (TNF) and IL-6, and help treat inflammatory diseases^61^. The IL-6 is a mediator that contributes to body defense and is produced in response to infections and tissue damage. It stimulates the acute inflammation phase and helps hematopoiesis and immune function, promoting differentiation and proliferation of many non-immune cells^62^, which is considered a good response, helping achieve GIT mucosa homeostasis^63^. On the other hand, when the gut microbiome is disrupted due to stress (weaning, transport, and disease), changes in feed intake, dehydration, or use of oral antimicrobials, a dysbiosis occurs, leading to an increase of inflammatory proteins such as IL-6 and TNF^7,64^. When this dysbiosis is more extensive leading, it leads to severe inflammation and a condition called “leaky gut”, where the tight junctions of the enterocytes are not functioning well, leading to a leak of intestinal bacterial to the bloodstream, causing the liver to switch to a metabolic organ to an immune organ, causing a decrease of the animal’s growth and performance^63,65^. Thus, developing an adaptive immune system is coordinated by gut microbiota and is important for disease resistance^6,7^. Also, the increase of butyrate induces a good response and inhibits inflammatory responses, inducing the immature T cells to convert to Treg cells, blocking inflammatory cells, and producing IL-10, which will turn on secretory IgA production and other antibacterial peptides, helping the GIT defense mechanism^63^. However, animals under chronic or intense stress tend to have differences in immune cell counts with higher eosinophils and lower levels of leukocytes and IL-6.

We did find differences in this trial for leukocyte counts. However, we have already observed that supplemented animals had a higher leukocyte count^22^. This corroborates with other finds where animals submitted to stress had lower leukocyte count^66^, evidencing the immunological effect of the EO supplementation. As for eosinophils, they are granulocyte cells with the same phagocytic and metabolic functions as neutrophils, but with an important role in killing parasites and dealing with certain types of allergies^67^. The lower eosinophils for the BEO animals that continued through the weeks could also add more evidence of a positive immunological effect of the EO supplementation. It is also important to mention that eosinophils are responsible for local defense, are present in GIT and respiratory tissues, and play an important role in biological functions and maintenance of homeostasis^68^. Observing our results, we see that EO supplementation had a positive impact not only on GIT development but also on the respiratory tract. Previous work has shown a positive impact on plants' secondary components over eosinophil-mediated inflammation^69^. As for hematological parameters and neutrophils, they were within normal range for specie and age^70,71^.

Respiratory diseases and pulmonary consolidation areas were not measured in this trial. However, the CON group presented heavier lungs, which could be a vestige of previous respiratory disease and replacement of epithelium for connective tissue, thus a heavier and more dense tissue. This difference could correlate to the eosinophil's role in the respiratory tract regulating fibrin accumulation, healing, and remodeling of the organ^68^. Thus, the BEO animals could have better tissue healing, less oxidative stress, and less local inflammation^35^; therefore, lighter respiratory tract. Animals under chronic or intense stress tend to have differences in immune cell counts with higher eosinophils, lower levels of leukocytes and IL-6^66^. Since BEO animals in our previous work the presented lower differences for respiratory score within the evaluated weeks and higher leukocyte count^22^, the respiratory and blood results of the present work evidenced the immunological effect of the BEO supplementation.

Additionally, due to eosinophils and organ differences between groups, it was expected to find differences in cytokines and inflammatory responses. It is known that pro-inflammatory cytokines (TNF and IL-6) and anti-inflammatory cytokines (IL-10) tend to increase with age^7^. However, besides a slight increase over time, no differences were seen in our buffy coat analysis for IL-6 and IL-10 between days 30 and 60 of age, and no differences were found between treatments. This could be because samples were collected only four weeks apart.

As for ileum and colon IL-6 and IL-10 gene expression, although similar between treatments, we expected to find differences since there were histological differences in the ileum and higher butyrate content in cecum for BEO animals. We had the statistical power to test these variables, and there is evidence that gut microbiota interacts with and influences RNA changes and expression^72^. However, as also cited for the buffy coat results, we might have collected the samples at the wrong time point to find gene expression differences. This can also explain why there were no differences in the proliferation and stimulus of splenocytes. The spleen is also a large immune organ with various immune cells such as lymphocytes and macrophages^30^. Thus, it was expected that the supplemented calves would have boosted and been more responsive to LPS, PMA, and *E. coli* extract and induced innate immune response. However, since this response could be correlated with the capacity of IL-6 secretion, IL-6 is secreted during the acute phase, other interleukin associated with lymphoproliferation, no differences were found for this parameter^66^, and both results correlate with each other. The spleen cells in our trial were from 60-day-old calves, healthy and not submitted to acute stress. New experiments must be done with varying concentrations of EO and time of sample collection to verify the immunomodulation effects promoted by this supplementation. Health and pathological challenges should also be tested to detect significant differences in immunological responses, how the supplementation helps overcome neonatal diseases, the behavior of blood cells, gene expression around disease time, and specially microbiota behavior of calves supplemented with EO.

## Conclusion

Feeding pre-weaned bull calves with an EO in the MR may be a promising alternative to improve the calf’s gut development, especially the lower gut, as well as improve immunological cells' response to health challenges during early life. This experimental database improves the results of feeding EO to young calves through a liquid diet. Future work should be done to understand better and evaluate the impact of feeding different EO, optimum dosage, way of providing it, and the impact on the gut microbiota of young dairy calves.

## Supplementary Information

Supplementary Information.

## Data Availability

All data generated or analyzed during this study are included in its Supplementary Information files.

## Abbreviations

BEO: Blend of essential oils group
BHB: Beta-hydroxy butyric acid
BrEEC: *E. coli* Extract control
BW: Body weight
CON: Control group
CP: Crude protein
DEI: Digestible energy intake
DM: Dry matter
ECE: *E. coli* Extract
EE: Ether extract
EO: Essential oils
GEF: Energy fecal excretion
GEI: Gross energy intake
GEMR: Milk replacer gross energy
GER: Refusals gross energy
GES: Starter gross energy
GEU: Energetic losses from urine
GIT: Gastrointestinal tract
Hb: Hemoglobin
HG: Heart girth
LPS: *E. coli* Lipopolysaccharide extract
MCHC: Mean corpuscular hemoglobin concentration
MCV: Mean corpuscular value
MEI: Metabolizable energy intake
MI: Mitotic index
MR: Milk replacer
MW: Metabolizable weight
NFR: Nutrient feces recovery
NI: Nutrient intake
NLR: Neutrophils to lymphocytes ratio
NH3-N: Ammonia nitrogen
NU: Urinary nitrogen
OM: Organic matter
PCV: Packed cell volume
PLR: Platelet lymphocytes ratio
PMA: *Phorbol 12-myristate 13-acetate* Positive control of the proliferation
RBC: Red blood count
RH: Rump height
TNF: Tumor necrosis factor
VFA: Volatile fatty acids
WH: Whiters height
